# Bacteria-mediated cancer therapy: A versatile bio-sapper with translational potential

**DOI:** 10.3389/fonc.2022.980111

**Published:** 2022-10-07

**Authors:** Miao Luo, Xiaoyu Chen, Haojin Gao, Fan Yang, Jianxiang Chen, Yiting Qiao

**Affiliations:** ^1^ School of Pharmacy, Institute of Hepatology and Metabolic Diseases, Department of Hepatology, the Affiliated Hospital of Hangzhou Normal University, Hangzhou Normal University, Hangzhou, China; ^2^ Jinan Microecological Biomedicine Shandong Laboratory, Jinan, China; ^3^ The First Affiliated Hospital, Key Laboratory of Combined Multi-Organ Transplantation, Ministry of Public Health, Key Laboratory of Organ Transplantation of Zhejiang Province, School of Medicine, Zhejiang University, Hangzhou, China

**Keywords:** bacteria-mediated cancer therapy, pathogen-associated molecular pattern (PAMP), drug delivery, cancer immunotherapy, bacterial cytotoxicity

## Abstract

Bacteria are important symbionts for humans, which sustain substantial influences on our health. Interestingly, some bastrains have been identified to have therapeutic applications, notably for antitumor activity. Thereby, oncologists have developed various therapeutic models and investigated the potential antitumor mechanisms for bacteria-mediated cancer therapy (BCT). Even though BCT has a long history and exhibits remarkable therapeutic efficacy in pre-clinical animal models, its clinical translation still lags and requires further breakthroughs. This review aims to focus on the established strains of therapeutic bacteria and their antitumor mechanisms, including the stimulation of host immune responses, direct cytotoxicity, the interference on cellular signal transduction, extracellular matrix remodeling, neoangiogenesis, and metabolism, as well as vehicles for drug delivery and gene therapy. Moreover, a brief discussion is proposed regarding the important future directions for this fantastic research field of BCT at the end of this review.

## Therapeutic bacteria for cancer treatment

Humans and bacteria have a symbiotic relationship. In average, one human being contains approximately 30 trillion human cells and 39 trillion bacteria (1). Diverse microbial community flourish in our epidermal tissues, mucosal tissues and digestive system, regulating our physiological behaviors in sophisticated ways. For example, the notorious *Helicobacter pylori* has been proved to increase the risk of developing gastric cancer. With the advances in detection methods based on genome sequencing, residential bacteria are also identified in tissues which were conventionally considered “sterile”, including brains, placentas, kidneys and cancerous tissues of breast (2). Even though our knowledge about their roles in human health is still superficial, pioneering scientists and physicians have made great effort to utilize these microorganisms to fight against human cancer.

The modern concept of bacteria-mediated cancer therapy (BCT) originated from more than a century ago. A surgical oncologist, William Coley, tentatively treated cancer patients with a mixture of inactivated *Streptococcus pyogenes* and *Serratia marcescens* (Coley’s Toxins) in 1983. From that attempt, isolated bacterial strains replaced deliberate infections by pathogenic bacteria in unsterile conditions during the mysterious natural therapies aiming at suppressing cancerous cell growth with infectious bacteria before the emergence of chemotherapies and radiotherapies. Even though BCT has never been a mainstream clinical treatment option for cancer, mechanistic and translational studies have been continuously devoted to elucidate the mechanisms by which bacteria could influence the growth of solid cancer. More bacteria strains have been chosen with rational consideration of their unique biological properties, and further engineered with modern genetic tools to achieve better therapeutic efficacy and safety profile. Here, we listed those strains which have gone through or in the middle of clinical trials registered under the guideline of U.S. Food and Drug Administration (**Table 1**).

**Table 1:** The therapeutic bacteria strains finish or undergo clinical evaluation.

Category	Strain/gene	Type of cancer	No. of patients treated	Clinical phase	NCT identification	Reference/Recruitment Status
*Salmonella typhimurium*	*Salmonella typhimurium VNP20009*	Melanoma, renal cell carcinoma	25	Phase I		Published (3)
		Melanoma	4	Phase I		Published (4)
		Refractory, superficial solid tumors	12-40	Phase I	NCT00004216	Unpublished, completed
	*Salmonella typhimurium TAPET-CD* *(VNP20009 expressing CD)*	Head & neck squamous cell carcinoma, esophageal adenocarcinoma	3			Published (5)
	*SalpIL2* *(Salmonella χ4550 expressing IL-2)*	Liver metastases of solid tumors	22	Phase I	NCT01099631	Unpublished, completed
	*S. typhiTy21/Anti-VEGFR-2 (VXM01)*	Pancreatic cancer	30	Phase I		Published (6)
*Salmonella* spp.	*TXSVN vaccine derived from Salmonella* sp. *(CVD908ssb)*	Multiple Myeloma	24		NCT03762291	Unpublished, Recruiting
*Clostridium novyi*	*Clostridium novyi-NT*	Colorectal cancer	2	Phase I	NCT00358397.	Unpublished, terminated
		Solid tumor malignancies	5	Phase I	NCT01118819	Unpublished, terminated
		Solid tumor malignancies	24	Phase I	NCT01924689	Published (7)
		retroperitoneal leiomyosarcoma	1	Phase I		Published (8)
*Listeria monocytogenes*	*ANZ-100/CRS-100 (LADD)*	Pancreatic cancer, colorectoal cancer, and melanoma all with liver metastases	9	Phase I	NCT00327652.	Published (9)
	*CRS-207 (LADD)*	Pancreatic cancer, mesothelioma, ovarian cancer, non-small-cell lung cancer	17	Phase I	NCT00585845.	Published (9)
		Pancreatic cancer	90	Phase II	NCT01417000.	Published (10)
		Mesothelioma	60	Phase I	NCT01675765.	Published (11)
		Pancreatic cancer	303	Phase II	NCT02004262	Published (12)
		Ovarian, fallopian or peritoneal cancer	35	Phase I/II	NCT02575807.	Unpublished; terminated
		Mesothelioma	10	Phase II	NCT03175172.	Unpublished; terminated
		Gastric, gastroesophageal junction, or esophageal cancer	5	Phase II	NCT03122548.	Unpublished; terminated
	*ADXS11-001(Lm-LLO)*	Cervical Intraepithelial Neoplasia	81	Phase II	NCT01116245.	Unpublished; terminated
		Cervical cancer	54	Phase II	NCT01266460.	Published (13)
		HPV-16+, p16+OPSCC		Phase I	NCT01598792ISRCTN47069182	Unpublished; terminated
		HPV positive oropharyngeal squamous cell carcinoma	15	Phase II	NCT02002182.	Unpublished; active, not recruiting
		Anal cancer	11	Phase I/II	NCT01671488.	Unpublished; terminated
		HPV+ Cervical cancer	25	Phase I/II	NCT02164461.	Unpublished; completed
		Cervical or HPV+ Head & neck cancer	66	Phase I/II	NCT02291055.	Unpublished; unknown
		Anal or rectal cancer	51	Phase II	NCT02399813.	Unpublished; completed
		Cervical cancer	450	Phase III	NCT02853604.	Unpublished; active, not recruiting
		HPV+ Non-small cell lung carcinoma	124	Phase II	NCT02531854.	Unpublished; unknown
	*Lm -LLO-E7 (Lm - LLO)*	Cervical cancer	15	Phase I		Published (14)
	*ADXS-NEO (Lm-LLO)*	HER2 expressing solid tumors	12	Phase I/II	NCT02386501.	Unpublished; completed
		Melanoma, Colon cancer, head and neck cancer, non-small cell lung cancer, urothelial carcinoma	5	Phase I	NCT03265080.	Unpublished; active, not recruiting
	*ADXS31-142*	prostate cancer	51	Phase I/II	NCT02325557	Unpublished; unknown
	*ADU-623 (LADD)*	Astrocytic tumors	11	Phase I	NCT01967758	Unpublished; completed
	*JNJ-64041809*	Prostate Cancer	26	Phase I	NCT02625857	Unpublished; completed
	*JNJ-64041757*	Non-small cell lung cancer,	18	Phase I	NCT02592967	Unpublished;terminated
	*pLADD (LADD)*	Colorectal neoplasms	28	Phase I	NCT03189030.	Unpublished; terminated
*Enterococcus gallinarum*	*MRx0518*	Pancreatic Cancer	15	Phase I	NCT04193904	Unpublished; Recruiting
*Bifidobacterium longum*	*APS001F* *(B. longum expressing CD)*	Advanced and/or Metastatic Solid Tumors	75	Phase I/II	NCT01562626	Unpublished, suspended
	*bacTRL-IL-12*	Solid Tumours	5	Phase I	NCT04025307	Unpublished, suspended
*Clostridium butyricum*	*CBM 588 Probiotic Strain*	Kidney Cancer	30	Phase I	NCT03829111	Published (15)
		Hematopoietic and Lymphoid Cell Neoplasm	36	Phase I	NCT03922035	Unpublished, active, not recruiting
		Kidney Cancer	30	Phase I	NCT05122546	Unpublished, recruiting

**Figure 1:**
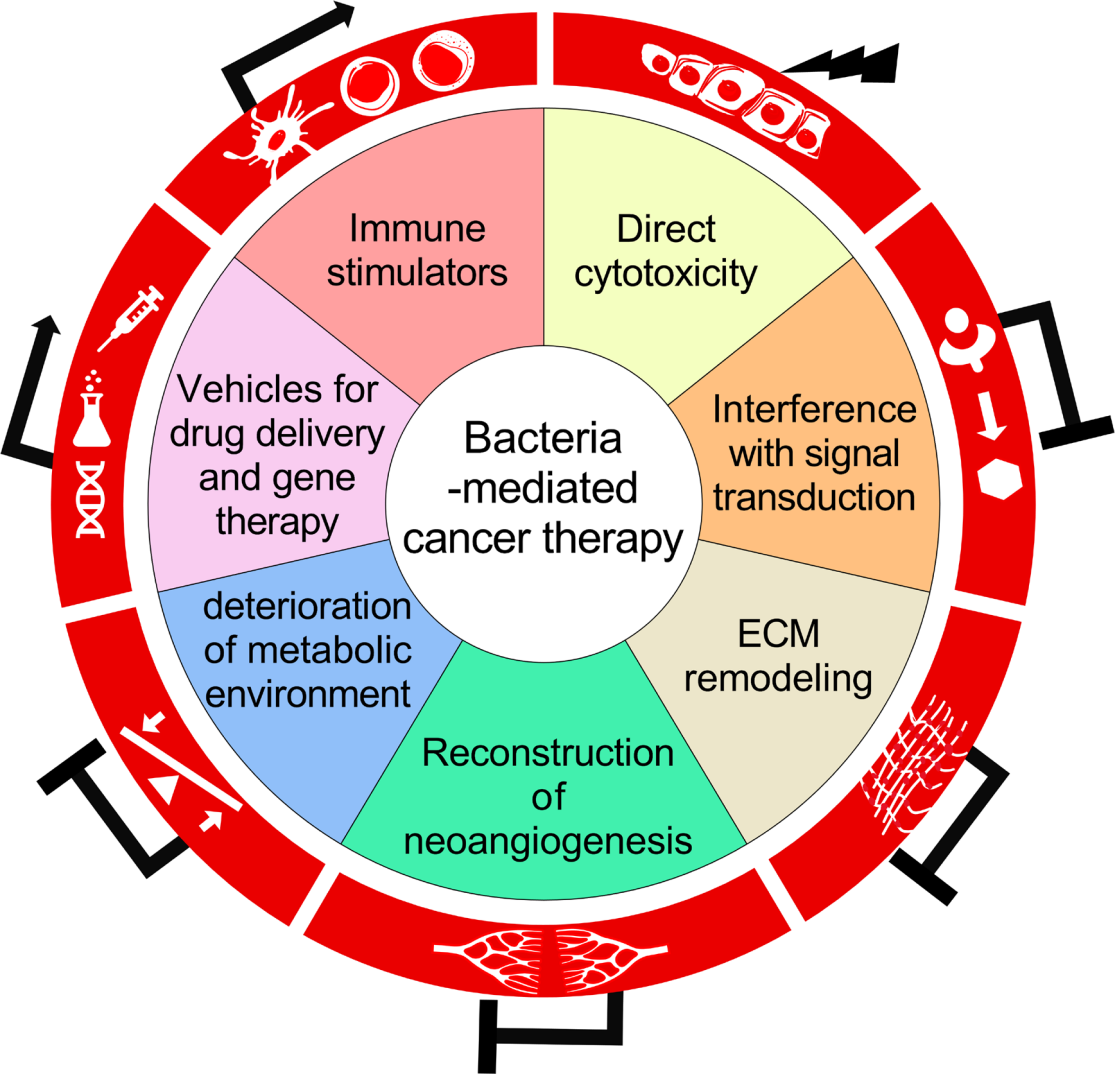
A schematic diagram summarizing the biological mechanisms for the antitumor activity of bacteria-mediated therapy.

This review would mainly discuss about the versatility of BCT, the underlying biological mechanisms in the triangular relationship among bacteria, cancer cells and host immune system, as well as problems ungently requiring thorough investigations in this field (**Figure 1**).

## The mechanisms of BCT

### Therapeutic bacteria function as immune stimulators

**Figure 2:**
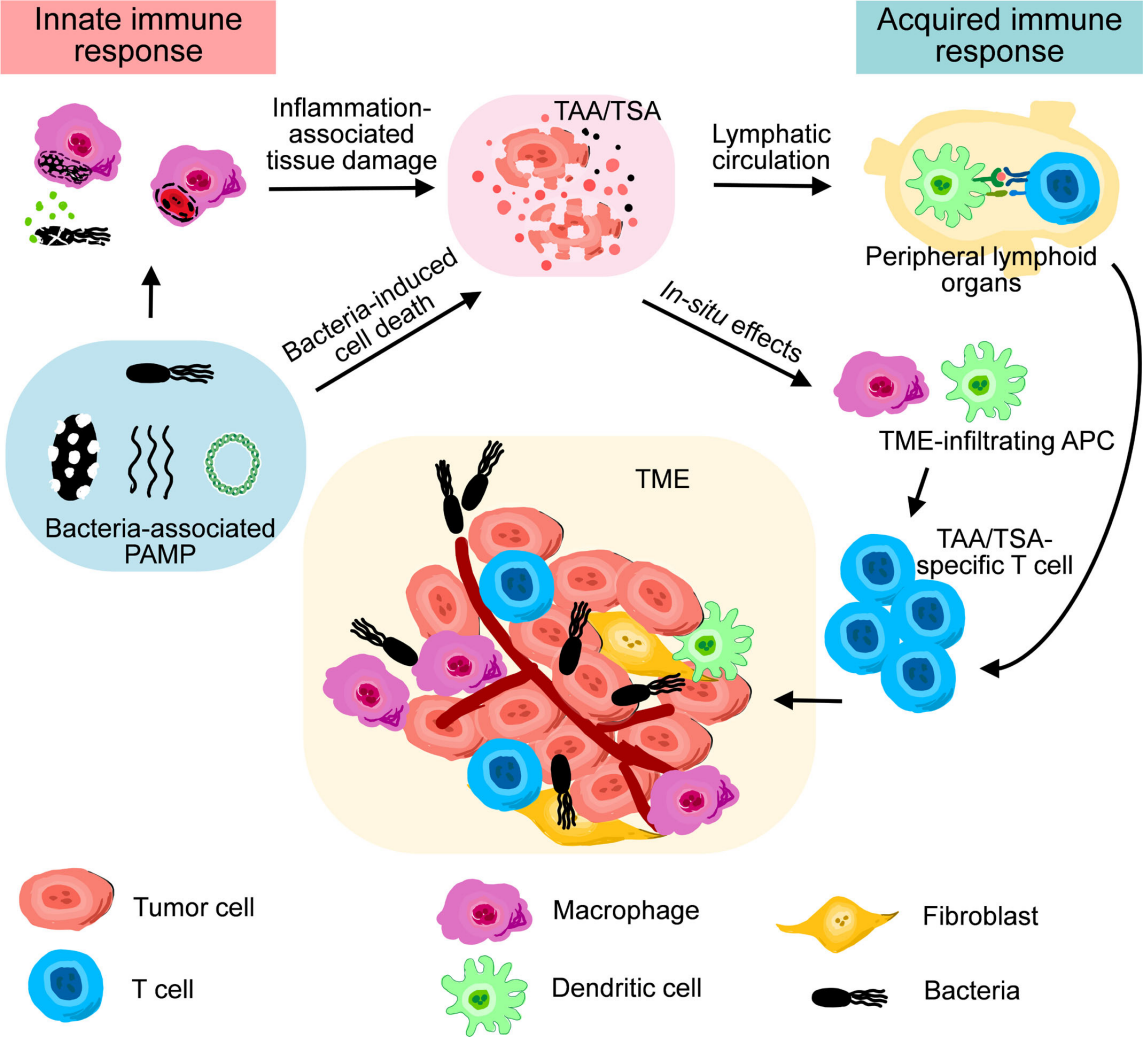
A schematic diagram illustrating the roles of innate immune response and acquired immune response during bacteria-mediated therapy.

Many bacterial components, such as lipopolysaccharide (LPS), flagella, and bacterial DNA, exhibits substantial and specific influences on the immunostimulatory responses induced by the administration of either live or inactivated bacteriological preparations (16). These pathogen-associated molecular patterns (PAMPs) immediately initiate innate immune responses, marked by the accumulation of granulocytes and macrophages into the sties of infection, as well as a coordinated elevation of proinflammatory cytokines and chemokines (17–19). During BCT in which aerobic or facultative anaerobic bacteria are used, a large titer of bacteria penetrates and colonizes in the hypoxic tumor microenvironment (TME) simultaneously. Such an intense infection, as well as consequent innate immune responses, concomitantly lead to the lysis of neighboring tumor cells and releasement of cellular content, including tumor-associated antigens (TAAs) and tumor-specific antigens (TSAs) (20). Antigen-presenting cells (APCs) engulf both bacteria and debris of cells infected by bacteria, and process them into antigens coupled with major histocompatibility complex (MHC) (21, 22). Eventually, antigen-specific T cells in draining lymph nodes are activated by APCs, marking the initiation of acquired immune responses. During the phase of acquired immune responses, the present of bacterial PAMPs in TME is still beneficial to maintain the proinflammatory status of T cells and macrophages (23, 24). Therefore, bacterial PAMPs play key functions for the antitumor effects of therapeutic bacteria (**Figure 2**).

LPS is an important structural component of the outer wall of Gram-negative bacteria. Structure of LPS consists of lipid A, the core oligosaccharide and the O-antigen. LPS forms a dense network of hydrophobic compounds through ionic and covalent bonds, providing barrier protection for bacterial outer membrane and protecting bacteria from the lethal effects of the complement system and macrophages (25). In addition to its role in bacterial membrane, LPS, especially its lipid A portion, is also an important signaling molecule for the innate immune system (26). LPS specifically activates Toll-like receptor 4 (TLR4) and CD14, which are widely expressed by CD8^+^ T cells, macrophages, natural killer (NK) cells and Dendritic cells (DCs), and initiates tumor-suppressive downstream signaling cascades mainly through myeloid differentiation primary response 88 (MYD88), such as the secretion of proinflammatory cytokines and cytotoxic factors, as well as the formation of inflammasome. For example, bacterial lipopolysaccharide (LPS) enhances prostate cancer metastasis potentially through NF-κB activation (27). *Salmonella* LPS could induce tumor-specific CD8^+^ T cell responses and the elevation of tumor necrosis factor-α (TNFα) in both TME and peripheral blood (28, 29). Moreover, Thuy Xuan Phan et al. demonstrated that *Salmonella typhimurium* enhanced inflammasome activation in tumor-infiltrating macrophages following their phagocytosis of *Salmonella*-infected tumor cells in a TLR4-dependent manner, and thus increased their level of interleukin-1β (IL-1β) secretion (30–32).

Flagellin is a subunit protein at the tip of bacterial flagella, the locomotive organ of bacteria, and it is recognized by TLR5 as a PAMP (33). Flagellin has been shown to induce perforin-dependent NK cell-mediated antitumor responses (34). Flagellin also activates NK cells through a TLR-independent pathway involving IL-18 and MYD88 to produce interferon-γ (IFNγ), a key cytokine in innate and adaptive immunity. Moreover, Francesc Coll et al. demonstrated that flagellin could significantly suppress tumor cell proliferation by decreasing the frequency of CD4^+^CD25^+^ regulatory T (Treg) cells (35). Chung Truong Nguyen *et al.* showed that flagellin also enhances a CD8^+^ T cell-dependent antitumor response in a peptide vaccine-based immunotherapeutic procedure (36).

Bacterial DNA is the genetic material shared by Gram-positive and Gram-negative bacteria, and it has broad-spectrum immune-stimulating effects. CpG motifs are the structural basis for its immune activity, which are recognized by TLR9 (37–40). Bacterial DNA directly activates mouse macrophages, B cells, and DCs in a TLR9-dependent manner, upregulates the expression of immunostimulatory molecules, modulates immune responses, and induces the secretion of various cytokines, including IL-12, IL-1, and TNFα (39, 41–44). Moreover, bacterial DNA indirectly activates Th1 and CTL through cytokines, DCs, and other monocytic cells (43–45). For example, KJ Stacey et al. showed that bacterial DNA directly activated APCs and upregulated the expression of MHC-class II molecules and costimulatory molecules such as CD86 and CD40, thus activating antigen-specific T cells to stimulate acquired immune responses (46, 47).

Apart from PAMPs, the production of TAAs/TSAs (tumor-associated antigens/tumor-specific antigens) is the key step for the successful shift from innate immune to acquired immune responses, which have the merit of high specificity and long-lasting memory (48). However, the efficacy of the concomitant lysis of tumor cells by bacteria-induced immune response is largely affected by the health conditions of recipients, such as the competency of immune system and the vasculature status of TME. Therefore, bacteria-mediated antigen delivery system is developed to bypass these limitations, in which bacteria are modified to carry TAA/TSAs by themselves (49, 50). This strategy of delivering TAAs/TSAs could effectively shape the host antitumor immune response and significantly suppress the growth of tumor. For example, Yu Mei et al. utilized the attenuated *Salmonella typhimurium* SL7207 to deliver a murine melanoma vaccine *via* the transformation of prokaryotic expression plasmids expressing the AIDA-1 autotransporter and DNA vaccine elements (51). Similarly, Xiong et al. constructed a novel SPI2-based oral *Salmonella* vaccine expressing mutant human *Survivin* in combination with NKT ligands, and achieved significantly improved generation of antigen-specific effector-memory CD8^+^ and CD4^+^ T cells, as well as increased antitumor efficacy, in murine models of colon cancer and glioblastoma (52). Apart from live bacteria-mediated antigen delivery system, bacteria components such as bacterial membrane could be directly used as vaccine adjuvant. For example, Ravi B Patel et al. used bacterial membrane-coated nanoparticles composed of immune activating PC7A/CpG polyplex core to *in situ* capture cancer neoantigens following radiation therapy, which facilitated their up-taken by DC cells to stimulate a strong antitumor T cell response (53).

## Therapeutic effects of BCT independent of immune responses

In addition to the induction of host immune responses, therapeutic bacteria exhibit direct cytotoxicity to cancer cells. An *in vitro* cell killing experiment showed that *Salmonella typhimurium* A1-R, an attenuated *Salmonella* strain, could cause cell death for prostate cancer cell lines *via* mixed cytotoxic mechanisms independent of immune responses (54). For example, *Salmonella* invasion induced rapid necrosis due to cytoplasmic swelling and membrane disruption (54). Extensive intracellular *Salmonella* duplication also led to the bursting of invaded cells (54). Moreover, *Clostridium novyi-NT* could secret phospholipases, hemolysins and lipases to disrupt the structure of lipid bilayers of cancer cells, thereby causing direct cytotoxicity (55, 56).

Apart from direct cytotoxicity, bacterial infection could suppress the growth of cancer cells through interfering with intracellular signal transduction. For example, *Salmonella typhimurium* flagellin could suppress the proliferation of breast cancer cells by activating the membrane-anchored TLR5 of cancer cells (57). *Salmonella* infection would also suppress AKT/mTOR pathway, thus leading to the induction of apoptotic and autophagic pathways (58). *Listeria monocytogenes* could kill tumor cells by enhancing cytosolic reactive oxygen species (ROS) levels through the activation of nicotinamide adenine dinucleotide phosphate oxidase and intracellular calcium mobilization, and such immunogenic tumor cell death would further activate CD8^+^ T cells to eliminate both primary tumors and metastases (59). Other than the proliferation and death of cancer cells, other cellular behaviors such as drug sensitivity would be influenced by therapeutic bacteria. For example, *Salmonella choleraesuis* induced a significant increase in the protein level of connexin 43 which mediated gap intercellular communication between cancer cells, thus sensitizing them to cisplatin (60).

The extracellular matrix (ECM) of tumor tissues would also be changed after the infection of therapeutic bacteria. ECM is an important component that maintains tissue integrity, and regulates cell behaviors through transmembrane signaling transduction (61). Solid tumors generally contain a high abundance of collagens and hyaluronic acids (HA), making them stiffer than normal tissues (62, 63). Malignant cells proliferate and migrate faster in such mechanical environment (64, 65). Meanwhile, accumulated ECM also creates structural obstacles physically for various cancer therapies, including chemotherapies and immunotherapies (66, 67). For example, collagen-laminin network forms a firm barrier for the diffusion of large molecules, while excessive HA dampens the passive release of drugs from blood vessels *via* increased osmotic pressure and viscosity of tissue fluid (68). Therapeutic bacteria could reduce the stiffness of ECM by multiple mechanisms. For example, *Salmonella typhimurium* could convert plasminogen to plasmin, a broad-spectrum serine protease, by inactivating Plasminogen activator inhibitor 1 (PAI-1), thus leading to the degradation of laminin as well as the activation of matrix metalloproteinases (MMPs) precursors to degrade collagens (69). *Listeria monocytogenes* are also capable to secrete MMPs (70). Some Gram-positive bacteria such as *Clostridium perfringens* (Mu toxin), *Clostridium difficile*, *Clostridium septicum* (γ toxin) and *Clostridium chauvoei* produces hyaluronidase that degrades HA (71–73). Other mechanisms by which bacteria lead to ECM disruption have been systematically reviewed by Lennert Steukers et al. (74). Taking advantages of these bacterial enzymes targeting intertumoral ECM, therapeutic bacteria have been utilized to combat solid tumors which are difficult to target by conventional chemotherapies. For example, Nancy D. Ebelt et al. recently reported that genetically modified attenuated *Salmonella typhimurium* expressing exogenously bacterial hyaluronidase could invaded into the desmoplastic tumors and degraded HA with a significantly enhanced efficacy in the orthotopic human pancreatic ductal adenocarcinoma mice models (75).

Besides cytotoxicity, signal transduction interference and ECM remodeling, the reconstruction of neoangiogenesis and metabolism landscape are also important features for bacteria-mediated tumor therapy. For example, *Salmonella typhimurium* VNP20009 treatment could reduce the formation of neovascularization, therefore leading to retarded diffusion of nutrients in TME (76, 77). *Salmonella choleraesuis* has been demonstrated as both tumoricidal and antiangiogenic for the treatment of hepatocellular carcinoma (78). Moreover, both *Salmonella typhimurium* and *Listeria monocytogenes* are glucose-consumers, which would increase nutritional stress for tumor cells and deteriorate the metabolic microenvironment in TME (79, 80).

## Therapeutic bacteria as tumor-targeting vehicles for drug delivery

Bacteria own several merits as pharmaceutical carriers for cancer therapies. First, anaerobic and facultative anaerobic bacteria have a natural tendency of penetrating and colonizing the hypoxic areas in tumor due to intrinsic locomotion, which exceeds the limits of vascularization. Second, bacteria own high surface-to-volume ratio, which provides suitable interface for particle absorption. Third, bacterial surface molecules such as polysaccharides and glycoproteins are potential subjects for chemical modifications. Last but not least, the cytotoxic effects of the delivered compounds and bacteria-induced immune activation might achieve synergistic effects during cancer therapy.

Therapeutic bacteria can be used as live vehicles (similar to “mules”). For example, Shuangqian Yan et al. reported a bacteria@MOFs system in which the flagellum of *Escherichia coli* MG1655 were modified with metal-organic framework (MOF) encapsulating chemical drugs through a one-step *in situ* method, could maintain viability and tumor selectivity to achieve effective delivery of chemical drugs to the poorly-vascularized areas inside the tumors (81). Moreover, additional guiding motifs could be incorporated. For example, Byung-Wook Park et al. linked *Escherichia coli* with drug-loaded polyelectrolyte multilayer microparticles with embedded magnetic nanoparticles at an approximately 1:1 ratio, to which system the authors referred as “microswimmers”, and such drug carriers with a diameter of 1 μm could directionally move at a mean speed of up to 22.5 μm/s under the orientation of an electromagnetic field, which would benefit the bacteria-mediated therapies for those highly vascularized tumors showing weak hypoxia (82). Packaging materials of different sizes and functions have been applied to decorate therapeutic bacteria (83–86), including polymeric particles (82, 87), polymer tubes (88), red blood cells (89–91), liposomes (92, 93), and nanoparticles (94–97). For example, Mukrime Birgul Akolpoglu et al. reported a biohybrid microrobotic platform by combining nanoliposomes and *E. coli* MG1655 (98). Recently, Fenton-like reactions which eliminate cancer cells by generating toxic ROS have developed rapidly as chemodynamic therapies, and therapeutic bacteria improve such therapies by biocatalyticly producing H_2_O_2_, a key reactant for Fenton-like reactions. For example, magnetic Fe_3_O_4_ nanoparticle-decorated *Escherichia coli* MG1655 overexpressing respiratory chain enzyme II (NDH-2) could support Fenton-like reaction by continuously producing H_2_O_2_, which triggered abundant tumor cell apoptosis by the excessive cytotoxic hydroxyl radicals (94).

However, frequent and repetitive administration is not suitable for live bacteria-mediated therapy, since its slow whole-body clearance might lead to accumulated systemic toxicity. Thus, drugs to be delivered by live bacteria should be chosen with great caution. Specifically, the ideal drugs to be delivered should be able to achieve sustained effects with one single dose.

Vesicles made with bacterial membranes, instead of live bacteria, are promising strategies to improve the safety profile of bacteria-mediated drug delivery. Small bacterial outer membrane vesicles (< 200 nm) are naturally released from Gram-negative bacteria during proliferation (99), and particles of such size often exhibit strong accumulation in TME through enhanced permeability and retention (EPR) effect during blood circulation. Recently Qi Chen et al. used DSPE-PEG-RGD-decorated bacterial outer membrane vesicles to encapsulate F127 mesoporous nanoparticles containing cytotoxic Tegafur. The obtained nanodrug could be preferentially accumulated at the site of tumors, and simultaneously induced the killing of tumor cells and the activation of immune cells such as macrophages and T cells. Eventually, the tumor antigens released by dead tumor cells initiated potent systemic immune responses eliminating both primary tumors and metastatic lesions (20).

Larger membrane vesicles maintaining the entire bacterial morphology can be produced *via* membrane perforation mediated by lysis gene *E* from bacteriophage øX174 (100), and they are often referred to as “bacterial ghost (BG)” by many researchers. BGs can be used immune adjuvants (101), and they have good structure integrity upon lyophilization, which is beneficial for massive production (102). Water-soluble drugs can be loaded into BGs through membrane poles (103), and these poles can be sealed by fusion with small membrane vesicles in the presence of Ca^2+^ ions to prevent drug leakage (104). *In vitro* evaluation showed that such drug-loaded bacterial ghosts could be up-taken by both macrophages and cancer cells, with a preference for macrophages (104). Considering the size of entire bacteria has exceed the range for strong EPR effect, BGs might not be the best choice for targeting tumor cells, but they are highly efficient for targeting APCs which preferentially engulf bacteria. For example, N.Dobrovolskienė et al. prepared BGs filled with tumor lysate containing numerous tumor antigens, and such antigen-loaded BGs induced a stronger dendritic cell activation compared to LPS (105).

## The combination of BCT and photothermal/photodynamic therapy

Photothermal therapy (PTT) and photodynamic therapy (PDT) are new strategies for cancer therapy. PTT uses photosensitizers (PSs) with specific light absorption to convert light energy into heat energy to eliminate cancer cells, while PDT uses PSs to produce excessive ROS in the present of specific wavelengths of light. PTT, as a non‐invasive cancer treatment, can cause strong tumor ablation and simultaneously induce heat shock proteins produced by tumor cells, which is a family of proteins with moderate immunostimulant function (106). In principle, PTT could also provide cancer treatment that causes relatively little damage to surrounding healthy tissue, since thermal effects only occur when near-infrared light is applied and only in the presence of PTT reagents (107). PDT takes advantage of the active metabolism of tumor tissue; whereby non-toxic photosensitizers accumulate in tumor tissue after injection. When the tumor tissue is irradiated with harmless visible light, the activated photosensitizer transfers its energy to surrounding intracellular oxygen that forms reactive oxygen species (ROS), which specifically destroy the tumor cells and neovascularization (108). PSs for PTT/PDT include organic dye molecules, organic nanoparticles, noble metal materials, carbon-based materials, quantum dots, and metal oxide nanoparticles (109–111). The *in vivo* distribution pattern and local drug concentration of PSs are critical factors determining the antitumor efficacy and systemic toxicity for PTT/PDT.

In bacteria-mediated PTT/PDT therapy, PSs are attached to the surface of bacteria to construct a bacteria-driven PSs delivery system, which significantly improves the tumor-targeting efficacy of PSs. For example, Chen et al. attached the INPs (PSs-containing indocyanine green (ICG)) to the surface of YB1 (an attenuated therapeutic *Salmonella Typhimurium* strain) *via* amide bonds, and the YB1-INPs complex achieved effective targeting hypoxic areas in tumor, thus eliminating bulk tumor tissues in mice after NIR laser irradiation. Meanwhile, YB1-INPs also exhibited satisfying fluorescence (FL) imaging ability (112). Moreover, many bacteria could immobilize environmental metal ions on cell surface through biomineralization process (113). Inspired by this phenomenon, Zhang’s group conjugated palladium nanoparticles (Pd NPs) on the surface of the facultative anaerobic bacterium *Shewanella oneidensis* MR-1 by biomineralization, and such bacteria-based photothermal therapeutical platform significantly increased photothermal capacity under near-infrared (NIR) laser irradiation (114). In addition to biomineralization, Cheng-Hung Luo et al. utilized cargo-carrying method and antibody-directed method to decorate anaerobic *Bifidobacterium breve* and *Clostridium difficile* with upconversion nanorods for tumor imaging and Au nanorods for photothermal ablation upon NIR excitation (115).

## The combination of BCT and gene therapy

In addition to chemotherapies, bacteria are effective carriers for gene therapies in the form of DNA and RNA. Plasmid is frequently used for bacteria-mediated gene therapies, due to its versatility for the engineering of genetic components, accessibility for massive production, high stability in physiology environment, as well as chemical activeness for further modifications. The gene products delivered by plasmids could be peptides, proteins, short hairpin RNAs and long non-coding RNAs. Both prokaryotic plasmids and eukaryotic plasmids can be delivered by bacteria, but their applications are different for cancer therapies.

Prokaryotic plasmids produce recombinant peptides and proteins within the bacterial protoplasm, and then these products are allocated to cytoplasm, membrane or extracellular space according to their sequence signature. Generally, the recombinant proteins produced by prokaryotic plasmids are released to TME either by secretion, bacterial lysis or endocytosis by multiple types of cells such as cancer cells, macrophages and granule cells (116, 117). For example, Loeffler et al. introduced the gene LIGHT, which encoded a human cytokine mediating tissue rejection, into attenuated *Salmonella typhimurium* by plasmid pGEN206, and the transformed *Salmonella* exhibited significantly improved antitumour activity without additional *in vitro* toxicity in murine carcinoma cell lines (118). Additionally, *Escherichia coli* stably transformed with a plasmid constitutively expressing nanobody antagonist against CD47 could stimulate systemic tumor antigen-specific immune responses, and induced durable tumor regression and long-term survival in a syngeneic tumor model (119). Such constitutive expressing systems are more suitable for the delivery of immune modulators compared to cytotoxin, since the short time window of uncontrolled bacterial distribution before they are restricted in TME could augment immune responses in favor of tumor elimination, while unintended delivery of cytotoxin to healthy organs would lead to systemic toxicity.

The incorporation of promoters in response to environmental signals, such as hypoxia, low pH and exogenous compounds, allows spatial and temporal regulation on the production of therapeutic molecules in TME. For example, hypoxia-inducible promoters, such as HIP-1 and NirB, are utilized to restrict gene expression within hypoxic TME, thus reducing toxicity to normal tissues (120–124). Meanwhile, chemical-inducible promoters (e.g., pBAD, pTet, and Pm) can activate gene expression through systemic administration of transcriptional inducers (e.g., L-arabinose, tetracyclines, and acetyl salicylic acid, respectively) *in vivo* (125–129).

In most cases, therapeutic proteins produced by bacteria need to be released into TME to convey biological effects (116). Therefore, the bacteria strains can be engineered to induce autolysis within TME, thus releasing therapeutic payloads in a controlled manner. For example, Eva María Camacho et al. developed an inducible autolysis system in response to anhydrotetracycline, in combination with a salicylate cascade system that allowed efficient production of therapeutic molecules in response to aspirin and a sifA mutation that liberated bacteria from the vacuoles to a cytosolic location for therapeutic *Salmonella* (130). Moreover, M. Omar Din et al. engineered *Salmonella Typhimurium* to lyse synchronously at a threshold population density and to release genetically encoded cargo in a pulsatile manner (131). Such engineering strategies may inspire development of therapeutic bio-communities within *in vivo* environments, where population dynamics are driven by interacting viruses, bacteria, and host immune cells (132).

Eukaryotic plasmids utilize the transcription and translation machinery of mammalian cells. Compared to prokaryotic systems, eukaryotic systems have the following advantages: firstly, protein products of eukaryotic systems are more biologically active due to the integrity of post-transcriptional modifications and higher structure, especially for those proteins with large molecular weight (133–135). In a study using *Salmonella Typhimurium* as a delivery vehicle, β-galactosidase expressed from a eukaryotic cassette induced substantially stronger immune responses than that expressed from a prokaryotic cassette (136). Secondly, the choice of therapeutic genes is more flexible, regardless of their subcellular locations of action. For example, RNA-based gene therapies often adopt eukaryotic plasmids to directly produce functional RNAs within target cells, thus bypassing the restrictions of RNA uptake efficacy *in vivo*. Huijie Jia et al. reported that attenuated *Salmonella Typhimurium* harboring eukaryotic plasmids expressing endostatin and Stat3-specific small interfering RNA (siRNA) conferred significant tumor-suppression effects in mouse tumor models (137). Moreover, therapeutic bacteria carrying eukaryotic plasmids encoding cytokines such as IL-4 and IL-18, and angiogenesis inhibitors such as endostatin and thrombospondin all resulted in retardation of tumor growth and prolonged survival of tumor-bearing mice (134, 138–140). Last but not least, eukaryotic plasmids could be loaded either within the protoplasm or on the surface of bacteria. Therefore, a considerable amount of plasmids can be loaded to a single bacterium due to surface-to-volume ratio (127).

However, the efficacy of plasmid endocytosis is the bottleneck for the eukaryotic plasmid-based therapies. Macrophages, the most important phagocytes in TME, are the ideal target for bacteria-mediated gene therapies. G Dietrich et al. reported that eukaryotic expression vectors can be delivered to macrophages using attenuated self-destructing *Listeria monocytogenes* (141). Similarly, Igentschev et al. also developed a plasmid-vector system targeting APCs with *Listeria monocytogenes*, which expressed the heterologous antigens under the control of an eukaryotic promoter in a similar fashion as in plasmids commonly used for vaccination with naked DNA (142). In addition to live bacteria, inactivated bacteria are also capable as vehicles of gene therapies targeting macrophages. For example, M.R.Miri et al. showed that BGs loaded with pEGFP-C1 plasmids were efficiently captured by murine macrophages (102, 143).

There are also some non-plasmid-dependent gene delivery methods. For example, Shuya Lu et al. reported that Chloroquine combined with PD-1 siRNA delivered with attenuated *Salmonella* could significantly enhance the tumor growth inhibition through upregulation of the number and activity of immune cells in tumor tissues (144). Qin Guo et al. developed a system in which bacterial outer membrane vesicles were coloaded with PTX and Redd1-siRNA (siRNA@M-/PTX-CA-OMVs) to regulate the tumor metabolic microenvironment and suppress tumor growth. Upon reaching the tumor site, the system was first triggered by tumor pH (pH 6.8) to release PTX. Then, the rest of the system would be taken up by M2 macrophages to increase their level of glycolysis (145).

Bacteria-mediated gene delivery is not only a therapeutic strategy, but also a useful diagnostic method. Genomically engineered bacterial can be detected non-invasively by fluoroscopy (146), magnetic resonance imaging (MRI) (147) and positron emission tomography (PET) (148) scan, which could be used to show the location of tumors. For example, *Escherichia coli* carrying pMW211-dsred plasmid could mark cancerous cells in their exact localization by luminescent signals (149). Studies have shown that there are many microorganisms differentially enriched in healthy population and cancer patients, such as gastric cancer, colorectal cancer, prostate cancer and oral cancer. Among them, many studies are focused on colorectal cancer and intestinal microorganisms, such as *Fusobacterium nucleatum*, *Peptostreptococcus stomatis* and *Streptococcus salivarius*, etc. All of these mentioned bacterial species are positively correlated with the occurrence of colorectal cancer and are expected to be potential diagnostic markers (150–152).

## Conclusion and perspective

In contrast with traditional cancer therapies such as chemotherapy and radiotherapy (153–156), which are less effective for the hypoxic tumors, therapeutic bacteria preferentially penetrate and colonize in hypoxic areas in the tumors. Therefore, it is beneficial to combine BCT with traditional cancer therapies. For example, the combination of *Salmonella typhimurium* with 5-fluorouracil inhibited MC26-LucF tumor growth and prolonged survival in mice (131). The combination of *Salmonella typhimurium* VNP20009 and Triptolide, a traditional Chinese medicine, showed significantly enhanced antitumor activity by modulating tumor angiogenesis and host immune response (77). Moreover, preclinical studies demonstrated that combined administration of Cytolysin A-expressing *Salmonella typhimurium* and radiation therapy could reduce tumor growth to a greater extent than bacterial monotherapy (157).

Apart from chemotherapy, radiation therapy is also commonly used for the treatment of solid tumors. While radiation therapy shows good efficacy for many types of cancer, its damage to surrounding normal tissues remains a difficult problem. A few studies regarding *E. coli, Salmonella Typhimurium, Salmonella, Clostridium* and other strains (158–161) have demonstrated that bacterial therapy combined with radiotherapy can reduce radiation-associated damage, improve the therapeutic effect in radiotherapy, and reduce metastasis (162). However, the mechanistic study on the combination of bacteria-mediated cancer therapy and radiotherapy is still superficial, and the behavior of cancer cells, bacteria, and especially immune cells during radiotherapy required more in-depth investigation.

However, the following questions remains to be solved for the research field of bacteria-mediated cancer therapy:

## The choice and optimization of bacterial strains

The clinical development of BCT faces substantial obstacles, mainly due to potential infection-related toxicity. For example, the Phase II clinical trial of ADXS11-001, an attenuated *Listeria monocytogenes* therapeutic vaccine, in patients with oropharyngeal cancer (NCT01598792) were suspended after a patient developed systemic listeriosis following vaccination (163).

The ideal strain should meet the following criteria: ① High tumor specificity. For example, a *Salmonella* strain displaying an integrin-binding RGD peptide on its outer membrane protein A (OmpA) showed a >1000-fold enrichment in the αvβ3 integrin-expressing U87MG and M21 xenografts compared to the control strain and an impressively enhanced antitumor activity in the MDA-MB-231 and MDA-MB-435 xenograft tumor models (164). ② High tumor-killing effect. Bacteria can be engineered to obtain enhanced anti-tumor activity by means such as gene elements coding for cytotoxic agents, as discuss in previous chapters in this review. ③ No/low systemic toxicity. LPS is one of the most potent TNFα stimulators and thus responsible for Gram-negative sepsis (165). Deletion of the *msbB* gene from *Salmonella* results in loss of myristoylation of lipid A, a critical component of LPS, and reduces its toxicity of by 10,000-fold. For example, VNP20009 with deletions of *purI* and *msbB*, two genes necessary for adenine and lipid A synthesis, respectively, have been safely administered to patients with metastatic melanoma and renal cell carcinoma in a phase I clinical study (3, 4). However, no antitumor effect was observed in patients treated with VNP20009, which might due be to the over-attenuation of bacteria (3, 166). Therefore, it should be noted that some of the virulence factors may also be responsible for the intrinsic antitumor activity of live bacteria. Whenever possible, attenuation should be achieved without substantially compromising the antitumor activity, unless the bacterial strain is used for the purpose of vaccination only.

## The choice of patients suitable for BCT

The standards for participant recruitments may greatly affect the outcomes of clinical trials (23). The risks and potential benefits must be considered carefully for first-in-human (FIH) trials. In general, only patients without any response to conventional therapies should be enrolled in clinical trials. Even though the bacteria are highly attenuated, the administration of live bacteria still poses a serious risk of infection. Therefore, the immune status and prior/concomitant therapies of patients should be evaluated sufficiently during the design of clinical trials (167). For example, immunocompromised patients who receive other immunotherapies simultaneously should be excluded (168). In addition, patients with foreign transplants such as artificial heart valves should be excluded, since foreign transplants may provide refuges for oncolytic bacteria to escape immune clearance and cause serious adverse reactions (168).

## The interaction between therapeutic bacteria and host bacterial flora

The long-term interaction of bacterial therapy on the normal flora of patients have not been investigated. It is not known yet whether therapeutic bacteria would hibernate in the locations of immune exemption, which might lead to unexpected health crisis in the long term. Moreover, the influence of host bacterial flora, as well as the antibiotic usage habits might substantially influence the effect of bacteria-mediated cancer therapies, which have not been studied comprehensively. Therefore, successful bacteria-mediated cancer therapies require interdisciplinary expertise, including oncologists, infectious disease specialists, immunologists and microbiologists.

Although many published studies on bacteria-based biotherapies have shown promising therapeutic effect in experimental models, its drawbacks are equally evident. Firstly, safety is the major concern due to the infectious nature of the bacteria. Secondly, limited drug loading efficiency is another challenge dampening the anticancer effect of bacteria. Thirdly, the manufacturing process of live bacteria is more complex than that of the small molecule anticancer drugs. Last but not least, when live bacteria could be used in a clinical settings, the potential impact on the environment would be also a concern that should be properly addressed (169, 170).

In conclusion, BCT is an emerging category of experimental cancer treatment, and what we’ve discovered might be the tip of an iceberg. From the first attempt of Coley’s strategy until today, great progress has been achieved. Thus, with more understanding of its mechanism, the bacteria, as well as bacteria-related therapeutics would become powerful weapons in the battle against cancers in the near future.

## Author contributions

ML, XC, HG and FY are responsible for the collection, collation, and writing of the manuscript. JC and YQ are responsible for the concept development, revision, and review of the manuscript. All authors contributed to the article and approved the submitted version.

## Funding

This work was supported by funding from Natural Science Foundation of Zhejiang Province (LR21H160001, LY21H160025), the Research Project of Jinan Microecological Biomedicine Shandong Laboratory (JNL-2022029C), “Pioneer” and “Leading Goose” R&D Program of Zhejiang Province (2021C03G2153004), National Natural Science Foundation of China (82072646, 81903143), and Start-up Grant of HZNU (4125C5021820470), Zhejiang Xinmiao (New-Shoot) Talent Project of China (2021R426071).

## Conflict of interest

The authors declare that the research was conducted in the absence of any commercial or financial relationships that could be construed as a potential conflict of interest.

## Publisher’s note

All claims expressed in this article are solely those of the authors and do not necessarily represent those of their affiliated organizations, or those of the publisher, the editors and the reviewers. Any product that may be evaluated in this article, or claim that may be made by its manufacturer, is not guaranteed or endorsed by the publisher.

## References

[1] Schwabe RF Jobin C. The microbiome and cancer. Nat Rev Cancer (2013) 13(11):800–12. doi: 10.1038/nrc3610 PMC3986062 24132111

[2] Nejman D Livyatan I Fuks G Gavert N Zwang Y Geller LT. The human tumor microbiome is composed of tumor type-specific intracellular bacteria. Science (2020) 368(6494):973–80. doi: 10.1126/science.aay9189 PMC7757858 32467386

[3] Toso JF Gill VJ Hwu P Marincola FM Restifo NP Schwartzentruber DJ. Phase I study of the intravenous administration of attenuated salmonella typhimurium to patients with metastatic melanoma. J Clin Oncol (2002) 20(1):142–52. doi: 10.1200/JCO.2002.20.1.142 PMC2064865 11773163

[4] Heimann DM Rosenberg SA. Continuous intravenous administration of live genetically modified salmonella typhimurium in patients with metastatic melanoma. J Immunother (2003) 26(2):179–80. doi: 10.1097/00002371-200303000-00011 PMC2656370 12616110

[5] Nemunaitis J Cunningham C Senzer N Kuhn J Cramm J Litz C. Pilot trial of genetically modified, attenuated salmonella expressing the e. Coli Cytosine Deaminase Gene Refractory Cancer Patients Cancer Gene Ther (2003) 10(10):737–44. doi: 10.1038/sj.cgt.7700634 14502226

[6] Schmitz-Winnenthal FH Hohmann N Niethammer AG Friedrich T Lubenau H Springer M. Anti-angiogenic activity of Vxm01, an oral T-cell vaccine against vegf receptor 2, in patients with advanced pancreatic cancer: A randomized, placebo-controlled, phase 1 trial. Oncoimmunology (2015) 4(4):e1001217. doi: 10.1080/2162402X.2014.1001217 26137397 PMC4485742

[7] Janku F Zhang HH Pezeshki A Goel S Murthy R Wang-Gillam A. Intratumoral injection of clostridium novyi-nt spores in patients with treatment-refractory advanced solid tumors. Clin Cancer Res (2021) 27(1):96–106. doi: 10.1158/1078-0432.CCR-20-2065 33046513

[8] Roberts NJ Zhang L Janku F Collins A Bai RY Staedtke V. Intratumoral injection of clostridium novyi-nt spores induces antitumor responses. Sci Transl Med (2014) 6(249):249ra111. doi: 10.1126/scitranslmed.3008982 PMC4399712 25122639

[9] Le DT Brockstedt DG Nir-Paz R Hampl J Mathur S Nemunaitis J. A live-attenuated listeria vaccine (Anz-100) and a live-attenuated listeria vaccine expressing mesothelin (Crs-207) for advanced cancers: Phase I studies of safety and immune induction. Clin Cancer Res (2012) 18(3):858–68. doi: 10.1158/1078-0432.CCR-11-2121 PMC3289408 22147941

[10] Le DT Wang-Gillam A Picozzi V Greten TF Crocenzi T Springett G. Safety and survival with gvax pancreas prime and listeria monocytogenes-expressing mesothelin (Crs-207) boost vaccines for metastatic pancreatic cancer. J Clin Oncol (2015) 33(12):1325–33. doi: 10.1200/JCO.2014.57.4244 PMC4397277 25584002

[11] Hassan R Alley E Kindler H Antonia S Jahan T Honarmand S. Clinical response of live-attenuated, listeria monocytogenes expressing mesothelin (Crs-207) with chemotherapy in patients with malignant pleural mesothelioma. Clin Cancer Res (2019) 25(19):5787–98. doi: 10.1158/1078-0432.CCR-19-0070 PMC8132300 31263030

[12] Le DT Picozzi VJ Ko AH Wainberg ZA Kindler H Wang-Gillam A. Results from a phase iib, randomized, multicenter study of gvax pancreas and crs-207 compared with chemotherapy in adults with previously treated metastatic pancreatic adenocarcinoma (Eclipse study). Clin Cancer Res (2019) 25(18):5493–502. doi: 10.1158/1078-0432.CCR-18-2992 PMC7376746 31126960

[13] Huh WK Brady WE Fracasso PM Dizon DS Powell MA Monk BJ. Phase ii study of axalimogene filolisbac (Adxs-hpv) for platinum-refractory cervical carcinoma: An nrg Oncology/Gynecologic oncology group study. Gynecol Oncol (2020) 158(3):562–9. doi: 10.1016/j.ygyno.2020.06.493 PMC7487015 32641240

[14] Maciag PC Radulovic S Rothman J. The first clinical use of a live-attenuated listeria monocytogenes vaccine: A phase I safety study of lm-Llo-E7 in patients with advanced carcinoma of the cervix. Vaccine (2009) 27(30):3975–83. doi: 10.1016/j.vaccine.2009.04.041 19389451

[15] Dizman N Meza L Bergerot P Alcantara M Dorff T Lyou Y. Nivolumab plus ipilimumab with or without live bacterial supplementation in metastatic renal cell carcinoma: A randomized phase 1 trial. Nat Med (2022) 28(4):704–12. doi: 10.1038/s41591-022-01694-6 PMC9018425 35228755

[16] Sfondrini L Rossini A Besusso D Merlo A Tagliabue E Menard S. Antitumor activity of the tlr-5 ligand flagellin in mouse models of cancer. J Immunol (2006) 176(11):6624–30. doi: 10.4049/jimmunol.176.11.6624 16709820

[17] Avogadri F Martinoli C Petrovska L Chiodoni C Transidico P Bronte V. Cancer immunotherapy based on killing of salmonella-infected tumor cells. Cancer Res (2005) 65(9):3920–7. doi: 10.1158/0008-5472.CAN-04-3002 15867392

[18] Gu WY Wang LH Wu YH Liu JP. Undo the brake of tumour immune tolerance with antibodies, peptide mimetics and small molecule compounds targeting pd-1/Pd-L1 checkpoint at different locations for acceleration of cytotoxic immunity to cancer cells. Clin Exp Pharmacol P (2019) 46(2):105–15. doi: 10.1111/1440-1681.13056 30565707

[19] Fang YY Kang YH Zou H Cheng XX Xie T Shi LY. Beta-elemene attenuates macrophage activation and proinflammatory factor production *Via* crosstalk with Wnt/Beta-catenin signaling pathway. Fitoterapia (2018) 124:92–102. doi: 10.1016/j.fitote.2017.10.015 29066299

[20] Chen Q Bai H Wu W Huang G Li Y Wu M. Bioengineering bacterial vesicle-coated polymeric nanomedicine for enhanced cancer immunotherapy and metastasis prevention. Nano Lett (2020) 20(1):11–21. doi: 10.1021/acs.nanolett.9b02182 31858807

[21] Eberl G Boneca IG. Bacteria and mamp-induced morphogenesis of the immune system. Curr Opin Immunol (2010) 22(4):448–54. doi: 10.1016/j.coi.2010.06.002 20580214

[22] Drozdz M Makuch S Cieniuch G Wozniak M Ziolkowski P. Obligate and facultative anaerobic bacteria in targeted cancer therapy: Current strategies and clinical applications. Life Sci (2020) 261:118296. doi: 10.1016/j.lfs.2020.118296 32822716

[23] Zhou S Gravekamp C Bermudes D Liu K. Tumour-targeting bacteria engineered to fight cancer. Nat Rev Cancer (2018) 18(12):727–43. doi: 10.1038/s41568-018-0070-z PMC6902869 30405213

[24] Liu XH Wang J. The nucleosome remodeling and deacetylase complex has prognostic significance and associates with immune microenvironment in skin cutaneous melanoma. Int Immunopharmacol (2020) 88:106887. doi: 10.1016/j.intimp.2020.106887 32799111

[25] Putker F Bos MP Tommassen J. Transport of lipopolysaccharide to the gram-negative bacterial cell surface. FEMS Microbiol Rev (2015) 39(6):985–1002. doi: 10.1093/femsre/fuv026 26038291

[26] Park BS Lee JO. Recognition of lipopolysaccharide pattern by Tlr4 complexes. Exp Mol Med (2013) 45:e66. doi: 10.1038/emm.2013.97 24310172 PMC3880462

[27] Jain S Dash P Minz AP Satpathi S Samal AG Behera PK. Lipopolysaccharide (Lps) enhances prostate cancer metastasis potentially through nf-kappab activation and recurrent dexamethasone administration fails to suppress it *in vivo* . Prostate (2019) 79(2):168–82. doi: 10.1002/pros.23722 30264470

[28] Beutler B Cerami A. The biology of Cachectin/Tnf–a primary mediator of the host response. Annu Rev Immunol (1989) 7:625–55. doi: 10.1146/annurev.iy.07.040189.003205 2540776

[29] Huang X Zhu J Jiang Y Xu C Lv Q Yu D. Su5416 attenuated lipopolysaccharide-induced acute lung injury in mice by modulating properties of vascular endothelial cells. Drug Des Devel Ther (2019) 13:1763–72. doi: 10.2147/DDDT.S188858 PMC6536715 31213766

[30] Darji A Guzman CA Gerstel B Wachholz P Timmis KN Wehland J. Oral somatic transgene vaccination using attenuated s. typhimurium. Cell (1997) 91(6):765–75. doi: 10.1016/s0092-8674(00)80465-1 9413986

[31] Cochlovius B Stassar MJ Schreurs MW Benner A Adema GJ. Oral DNA vaccination: Antigen uptake and presentation by dendritic cells elicits protective immunity. Immunol Lett (2002) 80(2):89–96. doi: 10.1016/s0165-2478(01)00313-3 11750039

[32] Phan TX Nguyen VH Duong MT Hong Y Choy HE Min JJ. Activation of inflammasome by attenuated salmonella typhimurium in bacteria-mediated cancer therapy. Microbiol Immunol (2015) 59(11):664–75. doi: 10.1111/1348-0421.12333 26500022

[33] Chen J Qiao Y Chen G Chang C Dong H Tang B. Salmonella flagella confer anti-tumor immunological effect *Via* activating Flagellin/Tlr5 signalling within tumor microenvironment. Acta Pharm Sin B (2021) 11(10):3165–77. doi: 10.1016/j.apsb.2021.04.019 PMC8546927 34729307

[34] Leigh ND Bian G Ding X Liu H Aygun-Sunar S Burdelya LG. A flagellin-derived toll-like receptor 5 agonist stimulates cytotoxic lymphocyte-mediated tumor immunity. PloS One (2014) 9(1):e85587. doi: 10.1371/journal.pone.0085587 24454895 PMC3891810

[35] Coll F Harrison EM Toleman MS Reuter S Raven KE Blane B. Longitudinal genomic surveillance of mrsa in the uk reveals transmission patterns in hospitals and the community. Sci Transl Med (2017) 9(413):eaak9745. doi: 10.1126/scitranslmed.aak9745 29070701 PMC5683347

[36] Nguyen CT Hong SH Sin JI Vu HV Jeong K Cho KO. Flagellin enhances tumor-specific Cd8(+) T cell immune responses through Tlr5 stimulation in a therapeutic cancer vaccine model. Vaccine (2013) 31(37):3879–87. doi: 10.1016/j.vaccine.2013.06.054 23831323

[37] Krieg AM. Cpg motifs in bacterial DNA and their immune effects. Annu Rev Immunol (2002) 20:709–60. doi: 10.1146/annurev.immunol.20.100301.064842 11861616

[38] Bauer S Kirschning CJ Hacker H Redecke V Hausmann S Akira S. Human Tlr9 confers responsiveness to bacterial DNA *Via* species-specific cpg motif recognition. Proc Natl Acad Sci U.S.A. (2001) 98(16):9237–42. doi: 10.1073/pnas.161293498 PMC55404 11470918

[39] Hemmi H Takeuchi O Kawai T Kaisho T Sato S Sanjo H. A toll-like receptor recognizes bacterial DNA. Nature (2000) 408(6813):740–5. doi: 10.1038/35047123 11130078

[40] Takeshita F Leifer CA Gursel I Ishii KJ Takeshita S Gursel M. Cutting edge: Role of toll-like receptor 9 in cpg DNA-induced activation of human cells. J Immunol (2001) 167(7):3555–8. doi: 10.4049/jimmunol.167.7.3555 11564765

[41] Weighardt H Feterowski C Veit M Rump M Wagner H Holzmann B. Increased resistance against acute polymicrobial sepsis in mice challenged with immunostimulatory cpg oligodeoxynucleotides is related to an enhanced innate effector cell response. J Immunol (2000) 165(8):4537–43. doi: 10.4049/jimmunol.165.8.4537 11035094

[42] Hartmann G Krieg AM. Mechanism and function of a newly identified cpg DNA motif in human primary b cells. J Immunol (2000) 164(2):944–53. doi: 10.4049/jimmunol.164.2.944 10623843

[43] Lipford GB Bendigs S Heeg K Wagner H. Poly-guanosine motifs costimulate antigen-reactive Cd8 T cells while bacterial cpg-DNA affect T-cell activation *Via* antigen-presenting cell-derived cytokines. Immunology (2000) 101(1):46–52. doi: 10.1046/j.1365-2567.2000.00077.x 11012752 PMC2327064

[44] Bauer M Redecke V Ellwart JW Scherer B Kremer JP Wagner H. Bacterial cpg-DNA triggers activation and maturation of human Cd11c-, Cd123+ dendritic cells. J Immunol (2001) 166(8):5000–7. doi: 10.4049/jimmunol.166.8.5000 11290780

[45] Liang H Reich CF Pisetsky DS Lipsky PE. The role of cell surface receptors in the activation of human b cells by phosphorothioate oligonucleotides. J Immunol (2000) 165(3):1438–45. doi: 10.4049/jimmunol.165.3.1438 10903748

[46] Stacey KJ Sester DP Sweet MJ Hume DA. Macrophage activation by immunostimulatory DNA. Curr Top Microbiol Immunol (2000) 247:41–58. doi: 10.1007/978-3-642-59672-8_3 10689778

[47] Bao YF Chen QJ Xie YS Tao Z Jin KH Chen SL. Ferulic acid attenuates oxidative DNA damage and inflammatory responses in microglia induced by Benzo(a)Pyrene. Int Immunopharmacol (2019) 77:105980. doi: 10.1016/j.intimp.2019.105980 31699670

[48] Stern C Kasnitz N Kocijancic D Trittel S Riese P Guzman CA. Induction of Cd4(+) and Cd8(+) anti-tumor effector T cell responses by bacteria mediated tumor therapy. Int J Cancer (2015) 137(8):2019–28. doi: 10.1002/ijc.29567 25868911

[49] Zhang Y Miwa S Zhang N Hoffman RM Zhao M. Tumor-targeting salmonella typhimurium A1-r arrests growth of breast-cancer brain metastasis. Oncotarget (2015) 6(5):2615–22. doi: 10.18632/oncotarget.2811 PMC4413605 25575815

[50] Alimoradi H Matikonda SS Gamble AB Giles GI Greish K. Hypoxia responsive drug delivery systems in tumor therapy. Curr Pharm Des (2016) 22(19):2808–20. doi: 10.2174/1381612822666160217130049 26898739

[51] Mei Y Zhao L Liu Y Gong H Song Y Lei L. Combining DNA vaccine and Aida-1 in attenuated salmonella activates tumor-specific Cd4(+) and Cd8(+) T-cell responses. Cancer Immunol Res (2017) 5(6):503–14. doi: 10.1158/2326-6066.CIR-16-0240-T 28468915

[52] Xiong G Husseiny MI Song L Erdreich-Epstein A Shackleford GM Seeger RC. Novel cancer vaccine based on genes of salmonella pathogenicity island 2. Int J Cancer (2010) 126(11):2622–34. doi: 10.1002/ijc.24957 PMC2993175 19824039

[53] Patel RB Ye M Carlson PM Jaquish A Zangl L Ma B. Development of an *in situ* cancer vaccine *Via* combinational radiation and bacterial-Membrane-Coated nanoparticles. Adv Mater (2019) 31(43):e1902626. doi: 10.1002/adma.201902626 31523868 PMC6810793

[54] Uchugonova A Zhang Y Salz R Liu F Suetsugu A Zhang L. Imaging the different mechanisms of prostate cancer cell-killing by tumor-targeting salmonella typhimurium A1-r. Anticancer Res (2015) 35(10):5225–9. 26408681

[55] Bettegowda C Huang X Lin J Cheong I Kohli M Szabo SA. The genome and transcriptomes of the anti-tumor agent clostridium novyi-nt. Nat Biotechnol (2006) 24(12):1573–80. doi: 10.1038/nbt1256 PMC9338427 17115055

[56] Li L You LS Mao LP Jin SH Chen XH Qian WB. Combing oncolytic adenovirus expressing beclin-1 with chemotherapy agent doxorubicin synergistically enhances cytotoxicity in human cml cells *in vitro* . Acta Pharmacol Sin (2018) 39(2):251–60. doi: 10.1038/aps.2017.100 PMC5800462 28905936

[57] Cai Z Sanchez A Shi Z Zhang T Liu M Zhang D. Activation of toll-like receptor 5 on breast cancer cells by flagellin suppresses cell proliferation and tumor growth. Cancer Res (2011) 71(7):2466–75. doi: 10.1158/0008-5472.CAN-10-1993 PMC3074302 21427357

[58] Lee CH Lin ST Liu JJ Chang WW Hsieh JL Wang WK. Salmonella induce autophagy in melanoma by the downregulation of Akt/Mtor pathway. Gene Ther (2014) 21(3):309–16. doi: 10.1038/gt.2013.86 24451116

[59] Kim SH Castro F Paterson Y Gravekamp C. High efficacy of a listeria-based vaccine against metastatic breast cancer reveals a dual mode of action. Cancer Res (2009) 69(14):5860–6. doi: 10.1158/0008-5472.CAN-08-4855 PMC3127451 19584282

[60] Chang WW Lai CH Chen MC Liu CF Kuan YD Lin ST. Salmonella enhance chemosensitivity in tumor through connexin 43 upregulation. Int J Cancer (2013) 133(8):1926–35. doi: 10.1002/ijc.28155 23558669

[61] Huang J Zhang L Wan D Zhou L Zheng S Lin S. Extracellular matrix and its therapeutic potential for cancer treatment. Signal Transduct Target Ther (2021) 6(1):153. doi: 10.1038/s41392-021-00544-0 33888679 PMC8062524

[62] Kalluri R. Basement membranes: Structure, assembly and role in tumour angiogenesis. Nat Rev Cancer (2003) 3(6):422–33. doi: 10.1038/nrc1094 12778132

[63] Insua-Rodriguez J Oskarsson T. The extracellular matrix in breast cancer. Adv Drug Delivery Rev (2016) 97:41–55. doi: 10.1016/j.addr.2015.12.017 26743193

[64] Paszek MJ Zahir N Johnson KR Lakins JN Rozenberg GI Gefen A. Tensional homeostasis and the malignant phenotype. Cancer Cell (2005) 8(3):241–54. doi: 10.1016/j.ccr.2005.08.010 16169468

[65] Alonso-Nocelo M Raimondo TM Vining KH Lopez-Lopez R de la Fuente M Mooney DJ. Matrix stiffness and tumor-associated macrophages modulate epithelial to mesenchymal transition of human adenocarcinoma cells. Biofabrication (2018) 10(3):035004. doi: 10.1088/1758-5090/aaafbc 29595143 PMC5904839

[66] Netti PA Berk DA Swartz MA Grodzinsky AJ Jain RK. Role of extracellular matrix assembly in interstitial transport in solid tumors. Cancer Res (2000) 60(9):2497–503. 10811131

[67] Rahbari NN Kedrin D Incio J Liu H Ho WW Nia HT. Anti-vegf therapy induces ecm remodeling and mechanical barriers to therapy in colorectal cancer liver metastases. Sci Transl Med (2016) 8(360):360ra135. doi: 10.1126/scitranslmed.aaf5219 PMC5457741 27733559

[68] Fan YF Shang WT Lu GH Guo KX Deng H Zhu XH. Decreasing hyaluronic acid combined with drug-loaded nanoprobes improve the delivery and efficacy of chemotherapeutic drugs for pancreatic cancer. Cancer Lett (2021) 523:1–9. doi: 10.1016/j.canlet.2021.09.016 34530049

[69] Haiko J Laakkonen L Juuti K Kalkkinen N Korhonen TK. The omptins of yersinia pestis and salmonella enterica cleave the reactive center loop of plasminogen activator inhibitor 1. J Bacteriol (2010) 192(18):4553–61. doi: 10.1128/JB.00458-10 PMC2937412 20639337

[70] Bitar AP Cao M Marquis H. The metalloprotease of listeria monocytogenes is activated by intramolecular autocatalysis. J Bacteriol (2008) 190(1):107–11. doi: 10.1128/JB.00852-07 PMC2223762 17965168

[71] Canard B Garnier T Saint-Joanis B Cole ST. Molecular genetic analysis of the nagh gene encoding a hyaluronidase of clostridium perfringens. Mol Gen Genet (1994) 243(2):215–24. doi: 10.1007/BF00280319 8177218

[72] Hafiz S Oakley CL. Clostridium difficile: Isolation and characteristics. J Med Microbiol (1976) 9(2):129–36. doi: 10.1099/00222615-9-2-129 933146

[73] Princewill TJ Oakley CL. Deoxyribonucleases and hyaluronidases of clostridium septicum and clostridium chauvoei. iii. relationship between the two organisms. Med Lab Sci (1976) 33(2):10–118. 940440

[74] Steukers L Glorieux S Vandekerckhove AP Favoreel HW Nauwynck HJ. Diverse microbial interactions with the basement membrane barrier. Trends Microbiol (2012) 20(3):147–55. doi: 10.1016/j.tim.2012.01.001 PMC7127156 22300759

[75] Ebelt ND Zuniga E Passi KB Sobocinski LJ Manuel ER. Hyaluronidase-expressing salmonella effectively targets tumor-associated hyaluronic acid in pancreatic ductal adenocarcinoma. Mol Cancer Ther (2020) 19(2):706–16. doi: 10.1158/1535-7163.MCT-19-0556 PMC7007852 31694889

[76] Jia LJ Wei DP Sun QM Jin GH Li SF Huang Y. Tumor-targeting salmonella typhimurium improves cyclophosphamide chemotherapy at maximum tolerated dose and low-dose metronomic regimens in a murine melanoma model. Int J Cancer (2007) 121(3):666–74. doi: 10.1002/ijc.22688 17397027

[77] Chen J Qiao Y Tang B Chen G Liu X Yang B. Modulation of salmonella tumor-colonization and intratumoral anti-angiogenesis by triptolide and its mechanism. Theranostics (2017) 7(8):2250–60. doi: 10.7150/thno.18816 PMC5505057 28740548

[78] Lee CH Wu CL Shiau AL. Salmonella choleraesuis as an anticancer agent in a syngeneic model of orthotopic hepatocellular carcinoma. Int J Cancer (2008) 122(4):930–5. doi: 10.1002/ijc.23047 17960612

[79] Bowden SD Rowley G Hinton JC Thompson A. Glucose and glycolysis are required for the successful infection of macrophages and mice by salmonella enterica serovar typhimurium. Infect Immun (2009) 77(7):3117–26. doi: 10.1128/IAI.00093-09 PMC2708584 19380470

[80] Grubmuller S Schauer K Goebel W Fuchs TM Eisenreich W. Analysis of carbon substrates used by listeria monocytogenes during growth in J774a.1 macrophages suggests a bipartite intracellular metabolism. Front Cell Infect Microbiol (2014) 4:156. doi: 10.3389/fcimb.2014.00156 25405102 PMC4217532

[81] Yan S Zeng X Wang Y Liu BF. Biomineralization of bacteria by a metal-organic framework for therapeutic delivery. Adv Healthc Mater (2020) 9(12):e2000046. doi: 10.1002/adhm.202000046 32400080

[82] Park BW Zhuang J Yasa O Sitti M. Multifunctional bacteria-driven microswimmers for targeted active drug delivery. ACS Nano (2017) 11(9):8910–23. doi: 10.1021/acsnano.7b03207 28873304

[83] Li J Ying S Ren H Dai J Zhang L Liang L. Molecular dynamics study on the encapsulation and release of anti-cancer drug doxorubicin by chitosan. Int J Pharm (2020) 580:119241. doi: 10.1016/j.ijpharm.2020.119241 32197982

[84] Feng C Ouyang J Tang ZM Kong N Liu Y Fu LY. Germanene-based theranostic materials for surgical adjuvant treatment: Inhibiting tumor recurrence and wound infection. Matter-Us (2020) 3(1):127–44. doi: 10.1016/j.matt.2020.04.022

[85] Kong N Zhang RN Wu GW Sui XB Wang JQ Kim NY. Intravesical delivery of Kdm6a-mrna *Via* mucoadhesive nanoparticles inhibits the metastasis of bladder cancer. P Natl Acad Sci USA (2022) 119(7):e2112696119. doi: 10.1073/pnas.2112696119 PMC8851555 35131941

[86] Ma HY Jiang ZM Xu JY Liu JQ Guo ZN. Targeted nano-delivery strategies for facilitating thrombolysis treatment in ischemic stroke. Drug Delivery (2021) 28(1):357–71. doi: 10.1080/10717544.2021.1879315 PMC8725844 33517820

[87] Akin D Sturgis J Ragheb K Sherman D Burkholder K Robinson JP. Bacteria-mediated delivery of nanoparticles and cargo into cells. Nat Nanotechnol (2007) 2(7):441–9. doi: 10.1038/nnano.2007.149 18654330

[88] Stanton MM Park BW Vilela D Bente K Faivre D Sitti M. Magnetotactic bacteria powered biohybrids target e. coli biofilms. ACS Nano (2017) 11(10):9968–78. doi: 10.1021/acsnano.7b04128 28933815

[89] Alapan Y Yasa O Schauer O Giltinan J Tabak AF Sourjik V. Soft erythrocyte-based bacterial microswimmers for cargo delivery. Sci Robot (2018) 3(17):eaar4423. doi: 10.1126/scirobotics.aar4423 33141741

[90] Buss N Yasa O Alapan Y Akolpoglu MB Sitti M. Nanoerythrosome-functionalized biohybrid microswimmers. APL Bioeng (2020) 4(2):026103. doi: 10.1063/1.5130670 32548539 PMC7141839

[91] Cao Z Cheng S Wang X Pang Y Liu J. Camouflaging bacteria by wrapping with cell membranes. Nat Commun (2019) 10(1):3452. doi: 10.1038/s41467-019-11390-8 31388002 PMC6684626

[92] Felfoul O Mohammadi M Taherkhani S de Lanauze D Zhong Xu Y Loghin D. Magneto-aerotactic bacteria deliver drug-containing nanoliposomes to tumour hypoxic regions. Nat Nanotechnol (2016) 11(11):941–7. doi: 10.1038/nnano.2016.137 PMC6094936 27525475

[93] Naciute M Kiwitt T Kemp RA Hook S. Bacteria biohybrid oral vaccines for colorectal cancer treatment reduce tumor growth and increase immune infiltration. Vaccine (2021) 39(39):5589–99. doi: 10.1016/j.vaccine.2021.08.028 34419301

[94] Fan JX Peng MY Wang H Zheng HR Liu ZL Li CX. Engineered bacterial bioreactor for tumor therapy *Via* fenton-like reaction with localized H2 O2 generation. Adv Mater (2019) 31(16):e1808278. doi: 10.1002/adma.201808278 30803049

[95] Zheng DW Chen Y Li ZH Xu L Li CX Li B. Optically-controlled bacterial metabolite for cancer therapy. Nat Commun (2018) 9(1):1680. doi: 10.1038/s41467-018-03233-9 29700283 PMC5920064

[96] Suh S Jo A Traore MA Zhan Y Coutermarsh-Ott SL Ringel-Scaia VM. Nanoscale bacteria-enabled autonomous drug delivery system (Nanobeads) enhances intratumoral transport of nanomedicine. Adv Sci (Weinh) (2019) 6(3):1801309. doi: 10.1002/advs.201801309 30775227 PMC6364498

[97] Huang Z Sun X Liu X Shen Y Wang K. Macrophages as an active tumour-targeting carrier of Sn38-nanoparticles for cancer therapy. J Drug Target (2018) 26(5-6):458–65. doi: 10.1080/1061186X.2017.1419359 29251524

[98] Akolpoglu MB Alapan Y Dogan NO Baltaci SF Yasa O Aybar Tural G. Magnetically steerable bacterial microrobots moving in 3d biological matrices for stimuli-responsive cargo delivery. Sci Adv (2022) 8(28):eabo6163. doi: 10.1126/sciadv.abo6163 35857516 PMC9286503

[99] Deatherage BL Cookson BT. Membrane vesicle release in bacteria, eukaryotes, and archaea: A conserved yet underappreciated aspect of microbial life. Infect Immun (2012) 80(6):1948–57. doi: 10.1128/IAI.06014-11 PMC3370574 22409932

[100] Montanaro J Inic-Kanada A Ladurner A Stein E Belij S Bintner N. Escherichia coli nissle 1917 bacterial ghosts retain crucial surface properties and express chlamydial antigen: An imaging study of a delivery system for the ocular surface. Drug Des Devel Ther (2015) 9:3741–54. doi: 10.2147/DDDT.S84370 PMC4516183 26229437

[101] Riedmann EM Kyd JM Cripps AW Lubitz W. Bacterial ghosts as adjuvant particles. Expert Rev Vaccines (2007) 6(2):241–53. doi: 10.1586/14760584.6.2.241 17408373

[102] Paukner S Kudela P Kohl G Schlapp T Friedrichs S Lubitz W. DNA-Loaded bacterial ghosts efficiently mediate reporter gene transfer and expression in macrophages. Mol Ther (2005) 11(2):215–23. doi: 10.1016/j.ymthe.2004.09.024 15668133

[103] Rabea S Alanazi FK Ashour AE Salem-Bekhit MM Yassin AS Moneib NA. Salmonella-innovative targeting carrier: Loading with doxorubicin for cancer treatment. Saudi Pharm J (2020) 28(10):1253–62. doi: 10.1016/j.jsps.2020.08.016 PMC7584810 33132719

[104] Paukner S Kohl G Jalava K Lubitz W. Sealed bacterial ghosts–novel targeting vehicles for advanced drug delivery of water-soluble substances. J Drug Target (2003) 11(3):151–61. doi: 10.1080/10611860310001593366 13129825

[105] Dobrovolskiene N Pasukoniene V Darinskas A Krasko JA Zilionyte K Mlynska A. Tumor lysate-loaded bacterial ghosts as a tool for optimized production of therapeutic dendritic cell-based cancer vaccines. Vaccine (2018) 36(29):4171–80. doi: 10.1016/j.vaccine.2018.06.016 29895501

[106] Huang X Pan J Xu F Shao B Wang Y Guo X. Bacteria-based cancer immunotherapy. Adv Sci (Weinh) (2021) 8(7):2003572. doi: 10.1002/advs.202003572 33854892 PMC8025040

[107] Jung HS Verwilst P Sharma A Shin J Sessler JL Kim JS. Organic molecule-based photothermal agents: An expanding photothermal therapy universe. Chem Soc Rev (2018) 47(7):2280–97. doi: 10.1039/c7cs00522a PMC5882556 29528360

[108] Fan Z Zhuang C Wang S Zhang Y. Photodynamic and photothermal therapy of hepatocellular carcinoma. Front Oncol (2021) 11:787780. doi: 10.3389/fonc.2021.787780 34950591 PMC8688153

[109] Li X Lovell JF Yoon J Chen X. Clinical development and potential of photothermal and photodynamic therapies for cancer. Nat Rev Clin Oncol (2020) 17(11):657–74. doi: 10.1038/s41571-020-0410-2 32699309

[110] Li J Zhao J Tan T Liu M Zeng Z Zeng Y. Nanoparticle drug delivery system for glioma and its efficacy improvement strategies: A comprehensive review. Int J Nanomedicine (2020) 15:2563–82. doi: 10.2147/IJN.S243223 PMC7173867 32368041

[111] Kong N Deng M Sun XN Chen YD Sui XB. Polydopamine-functionalized Ca-(Pcl-Ran-Pla) nanoparticles for target delivery of docetaxel and chemo-photothermal therapy of breast cancer. Front Pharmacol (2018) 9:125. doi: 10.3389/fphar.2018.00125 29527167 PMC5829531

[112] Chen F Zang Z Chen Z Cui L Chang Z Ma A. Nanophotosensitizer-engineered salmonella bacteria with hypoxia targeting and photothermal-assisted mutual bioaccumulation for solid tumor therapy. Biomaterials (2019) 214:119226. doi: 10.1016/j.biomaterials.2019.119226 31174068

[113] Huang J Lin L Sun D Chen H Yang D Li Q. Bio-inspired synthesis of metal nanomaterials and applications. Chem Soc Rev (2015) 44(17):6330–74. doi: 10.1039/c5cs00133a 26083903

[114] Chen QW Liu XH Fan JX Peng SY Wang JW Wang XN. Self-mineralized photothermal bacteria hybridizing with mitochondria-targeted metal–organic frameworks for augmenting photothermal tumor therapy. Advanced Funct Materials (2020) 30(14):1909806. doi: 10.1002/adfm.201909806

[115] Luo CH Huang CT Su CH Yeh CS. Bacteria-mediated hypoxia-specific delivery of nanoparticles for tumors imaging and therapy. Nano Lett (2016) 16(6):3493–9. doi: 10.1021/acs.nanolett.6b00262 27148804

[116] Russmann H Shams H Poblete F Fu Y Galan JE Donis RO. Delivery of epitopes by the salmonella type iii secretion system for vaccine development. Science (1998) 281(5376):565–8. doi: 10.1126/science.281.5376.565 9677200

[117] Walker BJ Stan GV Polizzi KM. Intracellular delivery of biologic therapeutics by bacterial secretion systems. Expert Rev Mol Med (2017) 19:e6. doi: 10.1017/erm.2017.7 28382885 PMC5479498

[118] Loeffler M Le'Negrate G Krajewska M Reed JC. Attenuated salmonella engineered to produce human cytokine light inhibit tumor growth. Proc Natl Acad Sci U.S.A. (2007) 104(31):12879–83. doi: 10.1073/pnas.0701959104 PMC1937560 17652173

[119] Chowdhury S Castro S Coker C Hinchliffe TE Arpaia N Danino T. Programmable bacteria induce durable tumor regression and systemic antitumor immunity. Nat Med (2019) 25(7):1057–63. doi: 10.1038/s41591-019-0498-z PMC6688650 31270504

[120] Mengesha A Dubois L Lambin P Landuyt W Chiu RK Wouters BG. Development of a flexible and potent hypoxia-inducible promoter for tumor-targeted gene expression in attenuated salmonella. Cancer Biol Ther (2006) 5(9):1120–8. doi: 10.4161/cbt.5.9.2951 16855381

[121] Yang YW Zhang CM Huang XJ Zhang XX Zhang LK Li JH. Tumor-targeted delivery of a c-terminally truncated fadd (N-fadd) significantly suppresses the B16f10 melanoma *Via* enhancing apoptosis. Sci Rep (2016) 6:34178. doi: 10.1038/srep34178 27767039 PMC5073321

[122] del Castillo FJ Leal SC Moreno F del Castillo I. The escherichia coli K-12 Shea gene encodes a 34-kda secreted haemolysin. Mol Microbiol (1997) 25(1):107–15. doi: 10.1046/j.1365-2958.1997.4391813.x 11902713

[123] Ludwig A Tengel C Bauer S Bubert A Benz R Mollenkopf HJ. Slya, a regulatory protein from salmonella typhimurium, induces a haemolytic and pore-forming protein in escherichia coli. Mol Gen Genet (1995) 249(5):474–86. doi: 10.1007/BF00290573 8544813

[124] Oscarsson J Mizunoe Y Uhlin BE Haydon DJ. Induction of haemolytic activity in escherichia coli by the slya gene product. Mol Microbiol (1996) 20(1):191–9. doi: 10.1111/j.1365-2958.1996.tb02500.x 8861216

[125] Jiang SN Park SH Lee HJ Zheng JH Kim HS Bom HS. Engineering of bacteria for the visualization of targeted delivery of a cytolytic anticancer agent. Mol Ther (2013) 21(11):1985–95. doi: 10.1038/mt.2013.183 PMC3831040 23922014

[126] Kim K Jeong JH Lim D Hong Y Lim HJ Kim GJ. L-asparaginase delivered by salmonella typhimurium suppresses solid tumors. Mol Ther Oncolytics (2015) 2:15007. doi: 10.1038/mto.2015.7 27119104 PMC4845971

[127] Royo JL Becker PD Camacho EM Cebolla A Link C Santero E. *In vivo* gene regulation in salmonella spp. by a salicylate-dependent control circuit. Nat Methods (2007) 4(11):937–42. doi: 10.1038/nmeth1107 17922017

[128] Cheng W Miao L Zhang H Yang O Ge H Li Y. Induction of interleukin 2 expression in the liver for the treatment of H22 hepatoma in mice. Dig Liver Dis (2013) 45(1):50–7. doi: 10.1016/j.dld.2012.08.014 22999060

[129] Loessner H Endmann A Leschner S Westphal K Rohde M Miloud T. Remote control of tumour-targeted salmonella enterica serovar typhimurium by the use of l-arabinose as inducer of bacterial gene expression *in vivo* . Cell Microbiol (2007) 9(6):1529–37. doi: 10.1111/j.1462-5822.2007.00890.x 17298393

[130] Camacho EM Mesa-Pereira B Medina C Flores A Santero E. Engineering salmonella as intracellular factory for effective killing of tumour cells. Sci Rep (2016) 6:30591. doi: 10.1038/srep30591 27464652 PMC4964584

[131] Din MO Danino T Prindle A Skalak M Selimkhanov J Allen K. Synchronized cycles of bacterial lysis for *in vivo* delivery. Nature (2016) 536(7614):81–5. doi: 10.1038/nature18930 PMC5048415 27437587

[132] Cheong I Huang X Bettegowda C Diaz LA Jr. Kinzler KW Zhou S. A bacterial protein enhances the release and efficacy of liposomal cancer drugs. Science (2006) 314(5803):1308–11. doi: 10.1126/science.1130651 17124324

[133] Hense M Domann E Krusch S Wachholz P Dittmar KE Rohde M. Eukaryotic expression plasmid transfer from the intracellular bacterium listeria monocytogenes to host cells. Cell Microbiol (2001) 3(9):599–609. doi: 10.1046/j.1462-5822.2001.00138.x 11553012

[134] Lee CH Wu CL Shiau AL. Systemic administration of attenuated salmonella choleraesuis carrying thrombospondin-1 gene leads to tumor-specific transgene expression, delayed tumor growth and prolonged survival in the murine melanoma model. Cancer Gene Ther (2005) 12(2):175–84. doi: 10.1038/sj.cgt.7700777 15375381

[135] Fu W Lan H Li S Han X Gao T Ren D. Synergistic antitumor efficacy of Suicide/Epnp gene and 6-methylpurine 2'-deoxyriboside *Via* salmonella against murine tumors. Cancer Gene Ther (2008) 15(7):474–84. doi: 10.1038/cgt.2008.19 18437183

[136] Darji A zur Lage S Garbe AI Chakraborty T Weiss S. Oral delivery of DNA vaccines using attenuated salmonella typhimurium as carrier. FEMS Immunol Med Microbiol (2000) 27(4):341–9. doi: 10.1111/j.1574-695X.2000.tb01448.x 10727890

[137] Jia H Li Y Zhao T Li X Hu J Yin D. Antitumor effects of Stat3-sirna and endostatin combined therapies, delivered by attenuated salmonella, on orthotopically implanted hepatocarcinoma. Cancer Immunol Immunother (2012) 61(11):1977–87. doi: 10.1007/s00262-012-1256-y PMC11028561 22527247

[138] Agorio C Schreiber F Sheppard M Mastroeni P Fernandez M Martinez MA. Live attenuated salmonella as a vector for oral cytokine gene therapy in melanoma. J Gene Med (2007) 9(5):416–23. doi: 10.1002/jgm.1023 17410612

[139] Liang K Liu Q Li P Han Y Bian X Tang Y. Endostatin gene therapy delivered by attenuated salmonella typhimurium in murine tumor models. Cancer Gene Ther (2018) 25(7-8):167–83. doi: 10.1038/s41417-018-0021-6 29755110

[140] Lee CH Wu CL Shiau AL. Endostatin gene therapy delivered by salmonella choleraesuis in murine tumor models. J Gene Med (2004) 6(12):1382–93. doi: 10.1002/jgm.626 15468191

[141] Dietrich G Bubert A Gentschev I Sokolovic Z Simm A Catic A. Delivery of antigen-encoding plasmid DNA into the cytosol of macrophages by attenuated suicide listeria monocytogenes. Nat Biotechnol (1998) 16(2):181–5. doi: 10.1038/nbt0298-181 9487527

[142] Gentschev I Dietrich G Spreng S Kolb-Maurer A Daniels J Hess J. Delivery of protein antigens and DNA by virulence-attenuated strains of salmonella typhimurium and listeria monocytogenes. J Biotechnol (2000) 83(1-2):19–26. doi: 10.1016/s0168-1656(00)00293-5 11000455

[143] Miri MR Behzad-Behbahani A Fardaei M Farhadi A Talebkhan Y Mohammadi M. Construction of bacterial ghosts for transfer and expression of a chimeric hepatitis c virus gene in macrophages. J Microbiol Methods (2015) 119:228–32. doi: 10.1016/j.mimet.2015.11.009 26578242

[144] Lu S Gao J Jia H Li Y Duan Y Song F. Pd-1-Sirna delivered by attenuated salmonella enhances the antitumor effect of chloroquine in colon cancer. Front Immunol (2021) 12:707991. doi: 10.3389/fimmu.2021.707991 34295341 PMC8290856

[145] Guo Q Li X Zhou W Chu Y Chen Q Zhang Y. Sequentially triggered bacterial outer membrane vesicles for macrophage metabolism modulation and tumor metastasis suppression. ACS Nano (2021) 15(8):13826–38. doi: 10.1021/acsnano.1c05613 34382768

[146] Yu YA Weibel S Szalay AA. Real-time imaging of tumors using replication-competent light-emitting microorganisms. Methods Mol Biol (2012) 872:159–75. doi: 10.1007/978-1-61779-797-2_11 22700410

[147] Alphandery E. Applications of magnetosomes synthesized by magnetotactic bacteria in medicine. Front Bioeng Biotechnol (2014) 2:5. doi: 10.3389/fbioe.2014.00005 25152880 PMC4126476

[148] Brader P Stritzker J Riedl CC Zanzonico P Cai S Burnazi EM. Escherichia coli nissle 1917 facilitates tumor detection by positron emission tomography and optical imaging. Clin Cancer Res (2008) 14(8):2295–302. doi: 10.1158/1078-0432.CCR-07-4254 18369089

[149] Sorensen M Lippuner C Kaiser T Misslitz A Aebischer T Bumann D. Rapidly maturing red fluorescent protein variants with strongly enhanced brightness in bacteria. FEBS Lett (2003) 552(2-3):110–4. doi: 10.1016/s0014-5793(03)00856-1 14527670

[150] Zeller G Tap J Voigt AY Sunagawa S Kultima JR Costea PI. Potential of fecal microbiota for early-stage detection of colorectal cancer. Mol Syst Biol (2014) 10:766. doi: 10.15252/msb.20145645 25432777 PMC4299606

[151] Eklof V Lofgren-Burstrom A Zingmark C Edin S Larsson P Karling P. Cancer-associated fecal microbial markers in colorectal cancer detection. Int J Cancer (2017) 141(12):2528–36. doi: 10.1002/ijc.31011 PMC5697688 28833079

[152] Mima K Nishihara R Qian ZR Cao Y Sukawa Y Nowak JA. Fusobacterium nucleatum in colorectal carcinoma tissue and patient prognosis. Gut (2016) 65(12):1973–80. doi: 10.1136/gutjnl-2015-310101 PMC4769120 26311717

[153] Wang XW Liu ZT Sui XB Wu QB Wang J Xu C. Elemene injection as adjunctive treatment to platinum-based chemotherapy in patients with stage Iii/Iv non-small cell lung cancer: A meta-analysis following the prisma guidelines. Phytomedicine (2019) 59:152787. doi: 10.1016/j.phymed.2018.12.010 31005810

[154] Lou JS Yao P Tsim KWK. Cancer treatment by using traditional Chinese medicine probing active compounds in anti-multidrug resistance during drug therapy. Curr Med Chem (2018) 25(38):5128–41. doi: 10.2174/0929867324666170920161922 28933300

[155] Cheng HB Ge XY Zhuo SQ Gao YA Zhu B Zhang JF. Beta-elemene synergizes with gefitinib to inhibit stem-like phenotypes and progression of lung cancer *Via* down-regulating Ezh2. Front Pharmacol (2018) 9:1413. doi: 10.3389/fphar.2018.01413 30555330 PMC6284059

[156] Li YC Sui XB Su ZQ Yu CY Shi XG Johnson NL. Meta-analysis of paclitaxel-based chemotherapy combined with traditional Chinese medicines for gastric cancer treatment. Front Pharmacol (2020) 11:132. doi: 10.3389/fphar.2020.00132 32174834 PMC7056897

[157] Liu X Jiang S Piao L Yuan F. Radiotherapy combined with an engineered salmonella typhimurium inhibits tumor growth in a mouse model of colon cancer. Exp Anim (2016) 65(4):413–8. doi: 10.1538/expanim.16-0033 PMC5111844 27301721

[158] Burdelya LG Krivokrysenko VI Tallant TC Strom E Gleiberman AS Gupta D. An agonist of toll-like receptor 5 has radioprotective activity in mouse and primate models. Science (2008) 320(5873):226–30. doi: 10.1126/science.1154986 PMC4322935 18403709

[159] Platt J Sodi S Kelley M Rockwell S Bermudes D Low KB. Antitumour effects of genetically engineered salmonella in combination with radiation. Eur J Cancer (2000) 36(18):2397–402. doi: 10.1016/s0959-8049(00)00336-1 11094316

[160] Wai SN Lindmark B Soderblom T Takade A Westermark M Oscarsson J. Vesicle-mediated export and assembly of pore-forming oligomers of the enterobacterial clya cytotoxin. Cell (2003) 115(1):25–35. doi: 10.1016/s0092-8674(03)00754-2 14532000

[161] Abdollahi H. Beneficial effects of cellular autofluorescence following ionization radiation: Hypothetical approaches for radiation protection and enhancing radiotherapy effectiveness. Med Hypotheses (2015) 84(3):194–8. doi: 10.1016/j.mehy.2014.12.021 25613566

[162] Bettegowda C Dang LH Abrams R Huso DL Dillehay L Cheong I. Overcoming the hypoxic barrier to radiation therapy with anaerobic bacteria. Proc Natl Acad Sci U.S.A. (2003) 100(25):15083–8. doi: 10.1073/pnas.2036598100 PMC299912 14657371

[163] Sacco JJ Evans M Harrington KJ Man S Powell N Shaw RJ. Systemic listeriosis following vaccination with the attenuated listeria monocytogenes therapeutic vaccine, Adxs11-001. Hum Vaccin Immunother (2016) 12(4):1085–6. doi: 10.1080/21645515.2015.1121338 PMC4962931 26618528

[164] Park SH Zheng JH Nguyen VH Jiang SN Kim DY Szardenings M. Rgd peptide cell-surface display enhances the targeting and therapeutic efficacy of attenuated salmonella-mediated cancer therapy. Theranostics (2016) 6(10):1672–82. doi: 10.7150/thno.16135 PMC4955065 27446500

[165] Beutler B Rietschel ET. Innate immune sensing and its roots: The story of endotoxin. Nat Rev Immunol (2003) 3(2):169–76. doi: 10.1038/nri1004 12563300

[166] Chorobik P Czaplicki D Ossysek K Bereta J. Salmonella and cancer: From pathogens to therapeutics. Acta Biochim Pol (2013) 60(3):285–97. 23828775

[167] Li XX Yin JY Tang J Li YH Yang QX Xiao ZY. Determining the balance between drug efficacy and safety by the network and biological system profile of its therapeutic target. Front Pharmacol (2018) 9:1245. doi: 10.3389/fphar.2018.01245 30429792 PMC6220079

[168] Chen Y Liu X Guo Y Wang J Zhang D Mei Y. Genetically engineered oncolytic bacteria as drug delivery systems for targeted cancer theranostics. Acta Biomater (2021) 124:72–87. doi: 10.1016/j.actbio.2021.02.006 33561563

[169] Lou X Chen Z He Z Sun M Sun J. Bacteria-mediated synergistic cancer therapy: Small microbiome has a big hope. Nanomicro Lett (2021) 13(1):37. doi: 10.1007/s40820-020-00560-9 34138211 PMC8187705

[170] Liu P Jia XZ Chen Y Yu Y Zhang K Lin YJ. Gut microbiota interacts with intrinsic brain activity of patients with amnestic mild cognitive impairment. CNS Neurosci Ther (2021) 27(2):163–73. doi: 10.1111/cns.13451 PMC7816203 32929861

